# Response of hemocytes profile in the black tiger shrimp (*Penaeus monodon*) against *Vibrio harveyi* induced by *Xylocarpus granatum* leaves extract

**DOI:** 10.14202/vetworld.2020.751-757

**Published:** 2020-04-22

**Authors:** Gina Saptiani, A. Syafei Sidik, Fikri Ardhani, Esti Handayani Hardi

**Affiliations:** 1 Laboratory of Aquatic microbiology, Faculty of Fisheries and Marine Sciences, Universitas Mulawarman. Jl. Gunung Tabur, Kampus Gunung Kelua, Samarinda 75124, East Kalimantan, Indonesia; 2 Laboratory of Experimental Pond, Faculty of Fisheries and Marine Sciences, Universitas Mulawarman. Jl. Gunung Tabur, Kampus Gunung Kelua, Samarinda 75124, East Kalimantan, Indonesia; 3 Department of Animal Science, Faculty of Agriculture, Universitas Mulawarman. Jl. Paser Balengkong, Kampus Gunung Kelua, Samarinda 75124, Indonesia

**Keywords:** hemocytes profile, mangrove plant extract, *Penaeus monodon*, *Vibrio harveyi*, *Xylocarpus granatum*

## Abstract

**Aim:**

The present study investigated hemocytes profile of black tiger shrimp (*Penaeus monodon*) induced with *Xylocarpus granatum* leaves extract to protect against *Vibrio harveyi* infection.

**Materials and Methods:**

*X. granatum* leaves were chopped into small size, air-dried, and extracted with one of the following solvents: Ethanol, distilled water, and seawater, whereas each solvent was given in three different concentrations (750 ppm, 1.000 ppm, and 1.250 ppm, respectively). Extracts were induced to 60 post-larvae shrimp in each treatment (three replicates, 20 shrimp for each) by immersing method and subsequently challenged with *V. harveyi*.

**Results:**

This study demonstrated different effectiveness among solvents used to extract *X. granatum* leaves, in which distilled water showed the most effective solvent as can be seen from the lowest percentage on anorexia, lethargic, and weakened reflex of shrimp compared with another solvent, positive and negative controls. Pathological symptoms for shrimp induced by *X. granatum* leaves extract were minimum with the highest survival rate compared with those of positive and negative control. Total hemocyte cells and its cell constituents such as semi-granular, granular, and hyaline cells on treatment group at 1.250 ppm were higher than controls.

**Conclusion:**

Leaves extract of *X. granatum* extract effectively inhibited *V. harveyi* infection, increased survival rate, and hemocytes cell of the experimental shrimp. Distilled water extract of *X. granatum* at 1.250 ppm demonstrated the highest protective effect toward *V. harveyi* infection on *P. monodon*.

## Introduction

Water quality and infectious diseases are major problems that influencing black tiger shrimp pond productivity in East Kalimantan, Indonesia. Water salinity and temperature in this area are susceptible to fluctuation [1]. This condition is known to have an immunosuppressive effect on shrimp [2]. A deterioration of pond environment is a key factor for pathogenic development that leading to disease outbreak [1]. Disease outbreak occurred during a week of post-larvae to a month of rearing period is largely associated with an increase of *Vibrio harveyi* population, a predominant *Vibrio* strain caused vibriosis in which often resulted in high mortality. Vibriosis is considered as failure causative agent in shrimp culture, whereas often leading to very significant economic losses [3,4]. Recently, Saptiani *et al*. [4] reported that *V. harveyi* is the most frequently detected bacterial species attacking shrimp pond culture in East Kalimantan, Indonesia. Mortality rates of larvae and post-larvae shrimp associated with *V. harveyi* infection were 40-75% at hatchery and 60-80% at pond culture.

Antibiotics and chemical compounds have long been used in aquaculture practice for controlling disease and mortality. However, intensive use of antibiotics is now a problem due to its numerous adverse effects on bacterial resistance and toxicity to the aquatic ecosystem [5]. In addition, vaccine has been used to prevent vibriosis in aquaculture. However, diversity of *Vibrio* strains becomes a limitation since vaccine is only effective for specific bacterial strain; thus, immune covering capacity is not optimal [6]. Over the past few decades, there has been a significant increase in natural compounds investigation as antibiotics replacer and therapeutic agents in aquatic animals [7]. The previous studies provided evidences that the incorporation of natural compounds such as plant extracts is effectively inhibiting fungal and bacterial growth in fish and shrimp culture [8-10]. Mangrove plants that grow around the coastal area of East Kalimantan Province are promising sources of natural bioactive compounds, but the exploration of potential as herbs in aquatic animals is limited. The previous identification showed that mangrove plants contain various secondary metabolites, such as alkaloids, flavonoids, phenolics, steroids and terpenoids, phenolic compounds, alkaloids, and flavonoids [11]. These compounds are well known to have antioxidant, antibacterial, antitumor, and antiviral activities [12]. Among mangrove plants, *Xylocarpus granatum* can be extracted as a source of natural bioactive compounds. The leaves have been used by coastal communities to cure injury and diarrhea since it has antibacterial and antidiarrheal effects while the fruit is used for skincare [13].

Since high mortality frequently occurred and it has become the major concern for shrimp farmers in East Kalimantan, research for combating disease problems is primarily important to promote sustainable development of shrimp aquaculture. Thus, the present study was aimed to examine the efficacy of *X. granatum* leaves extract on hemocyte cells response in black tiger shrimp that challenge with *V. harveyi*.

## Materials and Methods

### Ethical approval

All procedures performed in this study involving shrimp were approved by the Ethical Committee of Faculty of Fisheries and Marine Sciences, Universitas Mulawarman, Indonesia.

### Penaeus monodon

Black tiger shrimp (*P. monodon*) used in this experiment was PL20 strain obtained from Muara Badak hatchery farm, East Kalimantan, Indonesia. Shrimp larvae were originated from prawn spawned and reared under free antibiotics and chemical medicine water. The shrimp were *Vibrio* pathogenic free that had been tested by sampling and isolating the shrimp according to standard molecular technique. The shrimp had not been exposed to many diseases. Following this, the experimental shrimp were acclimatized for 2 days by randomly distributed into aquarium, whereas each containing 20 stocks.

### Preparation of *X. granatum* leaves extract

*X. granatum* were collected by the researchers in the shrimp pond area by putting the leaves randomly from the plants from Mahakam Delta in the area of Muara Badak, Kutai Kertanegara Regency, East Kalimantan Province, Indonesia. The extraction procedure was conducted according to Saptiani *et al*. [4]. In short, 300 g of leaves were macerated under 2.100 mL of different solvents, namely, ethanol 80%, distilled water, and 23% salinity of seawater for 24 h then followed by evaporation for extraction.

### *V. harveyi* culture

Culture of *V. harveyi* was a collection of the Laboratory of Aquatic Microbiology, Faculty of Fisheries and Marine Sciences, Universitas Mulawarman, Indonesia. Before infection, *V. harveyi* culture was tested for their pathogenicity by intramuscular injecting at 0.05 mL containing 10^4^ CFU/mL cells into five shrimp. After 5 days of infection, *V. harveyi* were isolated from hepatopancreas when the shrimp showing clinical symptoms. This infection procedure was repeated 3 times. Finally, *V. harveyi* were isolated and inoculated into Thiosulfate Citrate Bile Salta Sucrose Agar media and incubated at 33°C for 20 h followed by cell enumeration. Thereafter, bacterial culture on media plates was cultured into Tryptic Soy Broth (TSB) supplemented with 2% of NaCl (TSB salt) for challenge test.

### Water and aquarium

Water for experimental media was pathogenic free originated from seawater with 23% salinity. Water was precipitated in a tank for 2 days and transferred into an experimental aquarium and aerated. Water was precipitated in a tank for 2 days and transferred into an experimental aquarium and aerated. The total of water volume in each aquarium was maintained uniformly throughout the experimental period. Aquariums were set homogenously by maintaining the temperature at 28-29°C, pH at 7.0-7.5, and dissolved oxygen at 6.0-6.5 to ensure no effect of places, temperature, and light intensity. Water quality was also maintained by changing 25% of total volume daily to minimize ammonia, nitrite, and nitrate concentration.

### Experimental design

This research was performed in November 2019 for 21 days. Five treatments consisted of negative control (NaCl 0.85%), positive control (antibiotics=erythromycin 500 mg/1.000 mL), and three different solvents of *X. granatum* leaves extract (solvent 1=ethanol 80%, solvent 2=distilled water, and solvent 3=seawater with 23% salinity, respectively). There were three concentration levels for each solvent (1.250 ppm, 1.000 ppm, and 750 ppm, respectively), in which each treatment was replicated 3 times. In short, the extract was transferred into the aquarium followed by transferring 20 shrimp for each. On day 7, shrimp were subjected to *V. harveyi* challenge test by immersing the shrimp into 1 mL/3 L media containing 35.5×10^5^ CFU/mL pathogen.

### Analysis of clinical symptoms and pathology-anatomy of the shrimp

Clinical symptoms were observed daily by observing the lethargy, anorexia, and weakened reflex of the shrimp. The symptoms were calculated by counting the percentage of shrimp in each group that suspected to lethargy, anorexia, and weakened reflex signs. In addition, observation for pathology-anatomy (PA) was conducted at the end of the experimental period for the death shrimp. The PA observation was performed according to the changes in color, shape, consistency, and other abnormalities in the body and organs of shrimp, such as carapace, legs, tails, hepatopancreas, and stomach. The survival rate was determined by enumerating death shrimp daily and expressed as a percentage of final live shrimp to the initial number. For total hemocytes and differential hemocyte determination, hemolymph was withdrawn from the ventral sinus of individual shrimp into a syringe containing anticoagulant natrium thiosulfate in ratio 3:1. Thereafter, it was transferred to determine total hemocyte, differential hemocyte, and phagocytic by microscopy method.

Briefly, total hemocytes analysis was performed by withdrawn 0.1 mL hemolymph with a pipette and added with physiologist NaCl 0.85% solution up to 11 scale and homogeneous. Following this, the suspension was observed under colony counter Neuber to enumerate cell number. Further, differential hemocyte was determined by swapping the hemolymph onto glass object, air-dried, and added by methanol 95% for 5 min and then immersed into Giemsa 7% for 20 min. It was subsequently subjected to cell number enumeration [14].

The phagocytic activity analysis was carried out by mixture 0.1 mL hemolymph with *V. harveyi* culture (106 CFU/mL) in a microplate and incubated for 20 min. Following incubation, monolayer on glass object was air-dried and fixed with methanol for 5 min and continued with Giemsa 7% staining for 30 min. Thereafter, layers were washed by distilled water and air-dried then followed by cell enumeration under a microscope to determine phagocytic activity [14].

### Statistical analysis

Data for each parameter were analyzed descriptively according to a non-parametric analysis by comparing mean value within experimental treatments.

## Results and Discussion

### Clinical symptoms

Observation of clinical symptoms of shrimp challenged with *V. harveyi* was conducted during 21 days in aquarium. The initial physical appearance of shrimp under *X. granatum* leaves extract treatment and control was blue dark in color as their normal color. The body color was normal during 2-3 days exposure. Lethargic, swimming pattern, and anorexia were normal during this period.

On day 7, shrimp were subjected to *V. harveyi* challenge and the clinical symptoms were observed 2 days after infected. After infected, motion activity, anorexia, lethargic, and weak reflex response gradually decreased from day 9 to 14. These conditions steadily improved when the clinical conditions were evaluated on day 21, except for negative control that experienced the worst condition. According to Table-1, leaves extract of *X. granatum* effectively reduced the pathogenic effect from *V. harveyi* as indicated from lower percentage on lethargic, anorexia, and weakened reflexes. Among experimental treatments, shrimp induced with ethanol *X. granatum* leaves extract (XE) resulted in the lowest infection effect from *V. harveyi*.

**Table-1:** Mean value of the black tiger shrimp’s clinical symptoms induced with leaves extract of *Xylocarpus granatum* given in various concentrations (ppm).

Parameter	Treatment										

	Ethanol			Distilled water			Seawater			Control	
			
	750	1000	1250	750	1000	1250	750	1000	1250	C+	C−
Lethargy											
D-14	20.00	13.33	8.33	26.67	13.33	13.33	28.33	21.67	10.00	21.67	55.00
D-21	15.00	13.33	8.33	20.00	10.00	8.33	21.67	15.00	10.00	23.33	68.33
Anorexia											
D-14	26.67	20.00	15.00	28.33	20.00	21.67	35.00	26.67	20.00	28.33	60.00
D-21	23.33	20.00	10.00	21.67	20.00	15.00	28.33	25.00	20.00	28.33	75.00
Weakened reflexes											
D-14	21.67	6.67	6.67	23.33	15.00	13.33	33.33	20.00	18.33	31.67	55.00
D-21	11.67	3.33	6.67	18.33	11.67	13.33	23.33	20.00	10.00	30.00	71.67

C+=Positive control (antibiotics=erythromycin 500 mg/1.000 mL); C-=Negative control (NaCl 0.85%). D-14=Mean value observed on day 14; D-21=Mean value observed on day 21

Regarding on the effect of *X. granatum* extract, shrimp was less suffer compared to those of the control groups. The result also demonstrated different effectiveness among solvents used to extract *X. granatum* leaves as can be seen on the clinical symptoms, whereas distilled water showed the highest efficacy. Shrimp infected with *V. harveyi* appeared to have a significant decrease in anorexia, lethargic, and weakened reflex [11,14]. The clinical symptoms are presented in Table-1. The concentration of *X. granatum* extracts at 1.250 ppm showed higher efficacy in keeping animal from suffering as indicated by lower evidence of anorexia, weakened reflex, and lethargic. At the level 1.250 ppm, anorexia, weakened reflex, and lethargic decreased by 19.1%, 30.0%, and 23.5%, respectively. This result suggested that *X. granatum* leaves extract promoted immunostimulating effect for shrimp as shown by the better shrimp condition at the end of the experimental period (day 21). This result also confirmed that *X. granatum* leaves extract can inhibit *V. harveyi* infection on the black tiger shrimp.

Clinical observation also showed that the negative control group resulted in higher pathological and anatomical damages such as inflammation at their tail, feet, and the body in comparison with the treatment groups. Abnormalities were also found in the gill, hepatopancreas, and the stomach. These results were in line with the previous studies that infection of *V. harveyi* generates inflammatory symptoms such as change the body color, gill, and dark feet [15]. In addition, shrimp exposed to *V. harveyi* infection experienced a pathological and anatomical problem such as incomplete organs, reddish carapace, broken rostrum, reddish and broken legs, swollen and gripped uropod, brownish, reduced, disturbed hepatopancreas, and hard abdomen [11].

### Survival rate

**Figure-1:**
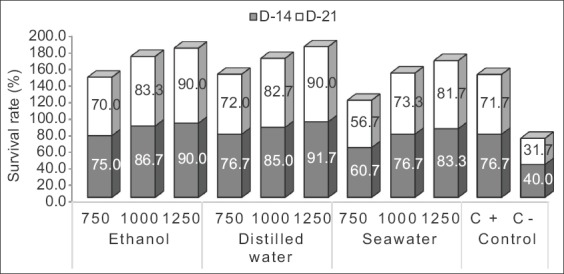
Mean value of the black tiger shrimp’s survival rate induced with leaves extract of *Xylocarpus granatum* given in various concentrations (ppm). C+=Positive control (antibiotics=Erythromycin 500 mg/1.000 mL); C−=Negative control (NaCl 0.85%). D-14=Mean value observed on day 14; D-21=Mean value observed on day 21.

Extract of *X. granatum* leaves in this research established a beneficial effect for enhancing the survival rate of the black tiger shrimp. It was reflected from Figure-1 that illustrated a higher survival rate on the group induced by *X. granatum* at 1.000-1.250 ppm. Treatment group at 1.250 ppm showed higher survival rate (91.67%) throughout the experimental period compared to those of positive and negative control (76.67% and 40%, respectively). These results confirmed that *X. granatum* leaves extract has an effective protection effect against *V. harveyi* infection, thus increased the survival rate of the black tiger shrimp.

*V. harveyi* is a luminescent bacterium that responsible for vibriosis outbreaks in East Kalimantan aquaculture in which significantly caused massive mortality, especially on shrimp pond culture. Many studies reported various strains of *Vibrio* that are present in the shrimp flock. As postulated by many researchers, *Vibrio* bacteria attack shrimp immunity in the deteriorated environment [16]. It has also been reported that vibriosis causes serious economic losses in hatchery farms [17]. A previous study showed that the survival rate of shrimp infected with *V. harveyi* was 53.62% [15].

### Hemocytes profile

The hemocytes profile of experimental treatments is shown in Table-2. Hemocyte cells analysis showed that shrimp induced by *X. granatum* at concentration 1.000-1.250 ppm were ranged around 13.20-20.20×10^5^ cells/mL (day 7). Following with challenge test, hemocyte cells increased to 14.50-20.50×10^5^ cells/mL (day 14) and then shifted to a decrease to 13.57-17.57×10^5^ cells/mL (day 21), in which this number is higher than control groups. Similar trend was also observed on the positive control group in which it was 12.60×10^5^ cells/mL on day 7, then increased to 14.10×10^5^ cells/mL on day 14 after being challenged and it further decreased to 8.67×10^5^ cells/mL on day 21. Total hemocytes of negative control shrimp on day 7 were 6.40×10^5^ cells/mL; then, it decreased to 5.67×10^5^ cells/mL and further decreased to 4.67×10^5^ cells/mL on day 21.

**Table-2:** Mean value of differential hemocytes of black tiger shrimp induced with leaves extract of *Xylocarpus granatum* given in various concentrations (ppm).

Parameter	Treatment										

	Ethanol			Distilled water			Seawater			Control	
			
	750	1000	1250	750	1000	1250	750	1000	1250	C+	C−
Total hemocytes											
D-7	11.33	15.33	17.67	10.67	17.67	20.00	10.33	13.33	17.00	12.67	6.67
D-14	9.67	16.67	16.67	9.62	16.67	20.40	9.79	14.67	18.02	14.33	5.80
D-21	11.33	16.67	15.67	11.33	15.67	17.67	10.67	13.67	15.67	8.67	4.67
Semi-granulocyte											
D-7	43.67	50.67	50.33	41.33	49.67	50.33	40.33	48.33	50.00	42.33	35.67
D-14	45.00	55.00	54.33	45.67	55.33	55.00	45.67	50.33	55.00	38.33	33.33
D-21	47.33	56.67	55.33	47.67	55.33	57.00	48.00	50.67	57.67	36.67	33.67
Granulocyte											
D-7	35.33	37.33	37.00	32.00	37.00	37.67	32.67	37.33	37.33	34.67	33.67
D-14	37.33	39.67	39.33	35.33	37.67	39.67	31.67	37.67	39.67	35.33	34.33
D-21	35.67	39.67	39.67	35.67	40.67	40.67	31.33	36.33	40.67	32.67	30.67
Hyaline											
D-7	21.00	12.00	12.67	26.67	13.33	12.00	27.00	14.33	12.67	23.00	30.67
D-14	17.67	5.33	6.33	19.00	7.00	5.33	22.67	12.00	5.33	26.33	32.33
D-21	17.00	3.67	5.00	16.67	4.00	2.33	20.67	13.00	1.67	30.67	35.67

C+=Positive control (antibiotics=erythromycin 500 mg/1.000 mL); C−=Negative control (NaCl 0.85%). D-7=Mean value observed on day 7; D-14=Mean value observed on day 14; D-21=Mean value observed on day 21

Hemocyte cells of the current findings were lower from the previous study, which reported total hemocyte cells of black tiger shrimp around 120-140×10^5^ cells/mL [18]. Raja *et al*. [19] also found a higher result that was 39.6±1.55×10^5^-163.8±1.05×10^5^ cells/mL. Total hemocyte cells of black tiger shrimp treated by the outer protein membrane of *Vibrio alginolyticus* cell wall increased to 31.75×10^5^ cells/mL [20].

Recently, phytogenic extracts have been extensively reported for their ability to improve shrimp immunity and disease resistance. From the current findings, *X. granatum* leaf extracts are considered to play an effective role in promoting shrimp immunity improvement shown by increasing hemocyte cells associated with *X. granatum* treatment, especially with distilled water solvent. Higher hemocyte cells obtained from *X. granatum* treatments than positive and negative control indicated that the extract has immunostimulant potential. On the other hand, the administration of Vitamins C, E, and β-1, 3-glucans carried out by AftabUddin *et al*. [21] showed an increase in shrimp total hemocyte cells by 15-18×10^6^ cells/mL. The total hemocyte of tiger prawns was 14×10^6^ cells/mL, after the challenge test was 11×10^6^ cells/mL, while the shrimp were given probiotics before the challenge test was 26×10^6^ cells/mL and after the challenge tests were 11×10^6^ cells/mL [22].

Mangrove plant *X. granatum* has been used by coastal communities for various purposes such as animal feed, food preservatives, and traditional herbal medicine. Mangroves plant species are promising sources of bioactive compounds that are acknowledged for their antimicrobial activity and antibiotics replacer against bacteria and fungi. Existing bioactive constituents in mangrove plant extracts can be used as herbs to treat various biological dysfunctions, with minimal risk effects and maximum benefit [23]. The use of plant extracts has been carried out by researchers worldwide as antibacterial and antifungal and can also be used as immunostimulants to prevent microbial resistance [4,24]. For centuries, medicinal plants have been used as immunostimulants, which increase the animal’s resistance to infection because they contain several chemical compounds that induce activation of immune cells [25].

In black tiger shrimp, circulating hemocytes are classified into semi-granular, granular, and hyaline cells. Differential hemocytes are a comparison among those types of hemocytes [26]. Hemocyte cells are involved in the immune defense system of shrimp through coagulation and melanin production through prophenoloxidase system. The granular and semi-granular cells serve in recognition and defense system. Leaves extract of *X. granatum* was established to enhance semi-granular cells of black tiger shrimp against *V. harveyi* protection. The immune response of shrimp increased as *X. granatum* increased that can be associated with continuously increasing semi-granular cells from 40.33-50.67% on day 7 to 47.33-57.67% on day 21. Conversely, there was a decrease in semi-granular cells of the positive control group from 42.33% to 36.67% after the challenge test. A similar decreasing trend also occurred in the negative control group.

Leaves extract of *X. granatum* also increased the granulocyte cells of tiger prawns on day 7 from 32.00-37.67% to 35.33-39.67% after the challenge test on day 14, except on shrimp treatment of seawater extract *X. granatum* at 750 ppm. Granulocyte cells of tiger prawns of positive control were 34.67% increased to 35.33% after exposed by *V. harveyi*. In the negative control group, granulocytes of tiger shrimp were 33.67% and increased to 34.33% after subjected to challenge tests. Immunostimulants agent is known to increase total hemocyte cells, granulocytes, semi-granulocytes, and hyaline cells [27].

### Phagocytosis profile

**Figure-2:**
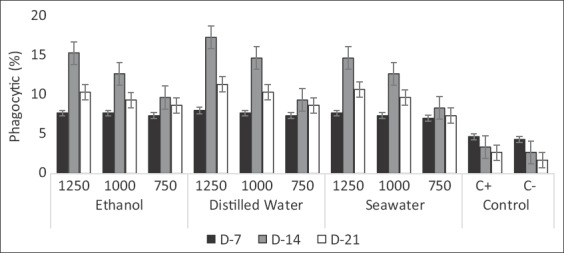
Mean value of phagocytic cells of the black tiger shrimp induced with leaves extract of *Xylocarpus granatum* given in various concentrations (ppm). C+=Positive control (antibiotics=Erythromycin 500 mg/1.000 mL); C−=Negative control (NaCl 0.85%). D-14=Mean value observed on day 14; D-21=Mean value observed on day 21.

Evaluation of phagocytic cells of black tiger shrimp induced with *X. granatum* extract is provided in Figure-2. Semi-granulocyte cells were superior to phagocyte bacterial activity in comparison to granular cells. According to Figure-2, phagocytic activities of shrimp received leaves extract of *X. granatum* were around 7.00-8.00%, while the positive control and negative control were 4.67% and 4.33%, respectively. Seven days after the challenge test, there was an increase in phagocytic activity to 8.33-17.33% in the hemocyte cell of shrimp that induced by *X. granatum* compared to the initial score. In contrast, phagocyte cells for positive and negative control decreased to 3.33% and 2.67%, respectively. At the end of the experimental period (day 21), phagocytosis decreased for groups that received *X. granatum* leaves extract although the value was still higher compared to those did not receive *X. granatum* leaves extract.

The current findings indicated that leaves extract of *X. granatum* can increase phagocytosis of shrimp hemocyte cell, especially after 7 days of challenge test. The highest phagocytosis was observed on distilled water extract at the level 1.250 ppm followed by ethanol extract at 1.250 ppm and then seawater extract at 1.250 ppm. According to Rengpipat *et al*. [22], phagocytosis ability of black tiger shrimp increased from 1.00% at initial observation to 6.00% after challenge test, while probiotic treatment showed a higher increase, from 2.20% to 10.50%. This result was also confirmed that immunostimulant supplementation is able to increase the phagocytic activity of shrimp hemocyte cell [28].

Mangrove flora *X. granatum* has been used as a medicinal plant by the coastal community because it has anti-diarrhea, antibacterial, antifilarial, cytotoxic, antiulcer, antidiabetic, antidyslipidemic, and cardiotonic activity [29]. Phagocytosis is one of the important roles of hemolymph in the invertebrate defense system and as an initial internal defense mechanism against foreign objects entering through the hemocyte circulation.

## Conclusion

Immersing the black tiger shrimp into leaves extract of *X. granatum* is effective in inhibiting *V. harveyi* infection. Anatomical and pathological symptoms for shrimp induced by leaves extract are minimum compared to those of positive and negative control; thus, the extract is effectively increased the survival rate. The immunostimulating effect was observed on *X. granatum*, shown by increasing total hemocyte cells and its cell constituents such as semi-granular, granular, and hyaline cells. This research also suggested that distilled water is the most effective solvent to extract *X. granatum* leaves because it produced leaves extract with the highest efficacy on hemocytes profile and minimum adverse effect on shrimp during observations. Optimum concentration of *X. granatum* was 1.250 ppm.

## Authors’ Contributions

The experimental design and conceptual research were implemented by GS and EHH. The experiment and laboratory analysis were performed by GS, ASS, and FA. GS and FA performed data analysis and data curation. The manuscript was written by GS, while FA, ASS, and EHH reviewed the manuscript. All authors contributed in giving input and approved the final manuscript.
